# New Insights on the Effects of Krill Oil Supplementation, a High-Fat Diet, and Aging on Hippocampal-Dependent Memory, Neuroinflammation, Synaptic Density, and Neurogenesis

**DOI:** 10.3390/ijms252111554

**Published:** 2024-10-28

**Authors:** John M. Andraka, Naveen Sharma, Yannick Marchalant

**Affiliations:** 1 Department of Physical Therapy, Central Michigan University, Mt. Pleasant, MI 48859, USA; 2 Neuroscience Program, Central Michigan University, Mt. Pleasant, MI 48859, USA; sharm2n@cmich.edu (N.S.); march1y@cmich.edu (Y.M.); 3 School of Health Sciences, Central Michigan University, Mt. Pleasant, MI 48859, USA; 4 Psychology Department, Central Michigan University, Mt. Pleasant, MI 48859, USA

**Keywords:** omega-3 fat, krill oil, brain, rodent, cytokines, cognition

## Abstract

Krill oil (KO) has been described as having the potential to ameliorate the detrimental consequences of a high-fat diet (HFD) on the aging brain, though the magnitude and mechanism of this benefit is unclear. We thus hypothesized that dietary KO supplementation could counteract the effects of cognitive aging and an HFD on spatial learning, neuroinflammation, neurogenesis, and synaptic density in the cortex and hippocampus of aged rats. Sixteen-month-old Sprague Dawley rats were fed for 12 weeks while being divided into four groups: control (CON); control with KO supplementation (CONKO); high-fat diet (HF); and high-fat diet with KO supplementation (HFKO). We measured food consumption, body mass, spatial memory (Morris water maze), microglia, neurogenesis, cytokine concentrations, and synaptic markers (post-synaptic density-95 and synaptophysin). Predictably, an HFD did induce significant differences in body weights, with the high-fat groups gaining more weight than the low-fat controls. However, KO supplementation did not produce significant changes in the other quantified parameters. Our results demonstrate that the dietary KO dose provided in the current study does not benefit hippocampal or cortical functions in an aging model. Our results provide a benchmark for future dosing protocols that may eventually prove to be beneficial.

## 1. Introduction

Reductions in synaptic density and hippocampal neurogenesis, as well as the presence of chronic, low-grade neuroinflammation, are detrimental processes that naturally occur during aging and following a poor diet [1,2,3,4,5]. While a combination of modifiable and nonmodifiable risk factors contribute to changes in brain health, the effects of normal aging and fatty foods are of particular concern considering increased life expectancy and a more obese population [6]. In fact, aging is the main risk factor for the development of cognitive decline and associated neurodegenerative diseases such as dementia [4]. Moreover, microglia, the principal resident central nervous system immune cells, become “sensitized” or “primed” in the aged brain, leading to the dysregulation of cytokine signaling proteins [4,7,8,9]. Specifically, chronically primed microglia promote a sustained, low-grade, pro-inflammatory brain environment which has negative consequences on long-term potentiation, and thus cognitive function, while increasing susceptibility to neurodegenerative disease processes [10,11,12,13]. Microglia are especially abundant in the hippocampus, cortex, and substantia nigra, and are highly dynamic in the adult brain, capable of proliferation in response to local pathogenic cues [14,15,16,17]. Undoubtedly, the capacity of microglia to migrate combined with a chronically activated morphological state contribute to excessive neuroinflammation and diminished synaptic plasticity in the cortex and hippocampus, which are key learning and memory structures in the brain [18]. Notably, diminished hippocampal neurogenesis (HN) and synaptic density are linked with aging [1,19,20]. Adult HN, especially in the dentate gyrus, supports learning and memory, while diminished capacity for regeneration has been associated with numerous cognitive and psychiatric disorders, including normal aging [3,21]. Indeed, HN declines with typical aging; however, this predictable reduction in neurogenesis appears to be worsened by the presence of neurodegenerative disease processes [1,22]. In addition to aging, HFD consumption is associated with a decline in neurogenesis and cognitive function [23,24,25,26,27,28]. Likewise, synaptic density and dendritic morphology are adversely affected, directly influencing neuronal plasticity, and are also closely linked with cognitive decline and Alzheimer’s disease [29,30,31]. Moreover, chronically activated or “primed” microglia are also associated with diminished synaptic density and memory deficits [18,28]. Overall, the combination of normal aging and fatty food consumption is linked with numerous deleterious changes to brain health, which require the exploration of novel preventative and therapeutic interventions [28].

Krill oil (KO), a marine-based, long-chain omega-3, consisting of polyunsaturated fatty acids (*n*-3 PUFAs), has been suggested as a supplement to counteract the undesirable effects of aging and an HFD on brain health and cognition, as it possesses promising anti-inflammatory and antioxidant neuroprotective properties [6,32]. Indeed, KO contains two vital long chain *n*-3 PUFAs, eicosapentaenoic acid (EPA; 20:5 *n*-3) and docosahexaenoic acid (DHA; 22:6 *n*-3), which are more easily digested and absorbed, and vital for cellular metabolism, enzymatic activity, and synaptic plasticity [33]. DHA and EPA have also shown potential for decreasing neuroinflammation, facilitating neurogenesis, supporting synaptic density, and improving cognitive functions [6,34,35,36,37,38]. Moreover, KO is biochemically unique compared to alternative sources of *n*-3, in that KO contains astaxanthin (ASTA) and choline [6]. ASTA naturally occurs in krill, is a potent antioxidant, and demonstrates the ability to counteract neuroinflammation, while choline has proven cognitive and neuroprotective benefits [35,39,40,41,42,43]. Despite its advantageous composition and purported health benefits, there is little evidence supporting the neuroprotective benefits of KO supplementation in the background of an HFD and aging. To our knowledge, no studies assessing memory have tested the benefits of KO in promoting neurogenesis or positively influencing synaptic density. Therefore, we hypothesized that dietary supplementation of KO, provided to aging rats fed an HFD, would benefit hippocampal-dependent memory, neuroinflammation, neurogenesis and synaptic density markers. This hypothesis was tested by administering a strict dietary protocol of varying amounts of saturated fats and KO supplementation to aged rats and then assessing spatial memory, inflammatory cytokine concentrations, synaptic plasticity associated proteins, microglial quantification, and hippocampal neurogenesis.

## 2. Results

### 2.1. Animals

Thirty-six Sprague Dawley rats were randomly assigned to four dietary groups. Five animals died during the 3 months of the dietary intervention and thus were excluded from all data analyses. Cause of death was not determined, though we concluded with our veterinary staff that it was likely due to normal aging. Deaths did not appear related to our protocol since they were somewhat evenly distributed between groups (2 CON, 2 HF, 1 HFKO). Mean age at termination was 19.0 months (SD ± 1.6), and there was no significant difference in age at termination between dietary groups, *p* = 0.552.

### 2.2. Food Consumption

**Figure 1:**
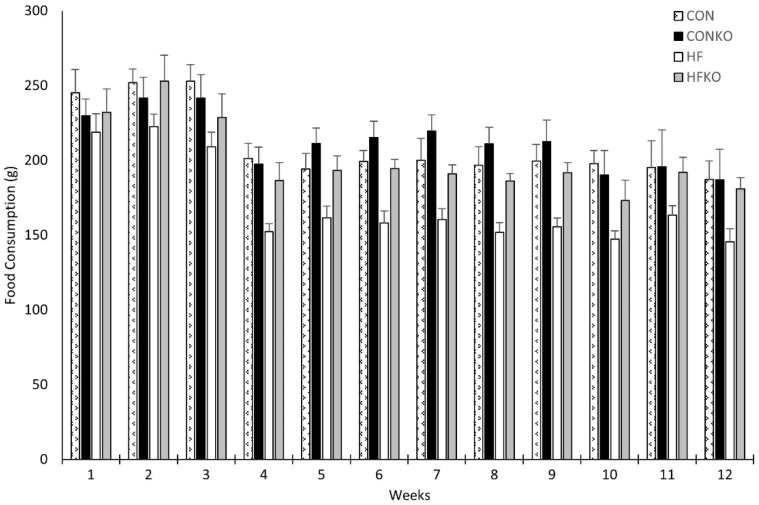
Comparison of weekly mean food consumption between dietary groups. Statistically significant between-subject effects were identified with lower food intake in the HF group when compared to both CON and HFKO groups (one-way repeated measures ANOVA, *p* < 0.05). Error bars represent SEM. n = 7–8 per group. CON: control diet; CONKO: control diet with krill oil supplementation; HF: high-fat diet; HFKO: high-fat diet with krill oil supplementation.

Food consumption was measured weekly (Figure 1). One-way repeated measures ANOVA identified statistically significant, between-subject differences in food consumption, *p* = 0.016. Food consumption was significantly lower in the HF group when compared to CON (*p* = 0.036) and HFKO (*p* = 0.048) determined by means of Tukey HSD post hoc analysis. No additional differences were identified between dietary groups. Considering average daily food consumption for both KO dietary groups, EPA and DHA supplementation was as follows: CONKO, 366.8 mg/kg/day and 170.3 mg/kg/day; HFKO, 293.6 mg/kg/day and 137.0 mg/kg/day, respectively.

### 2.3. Anthropometric Data

**Figure 2:**
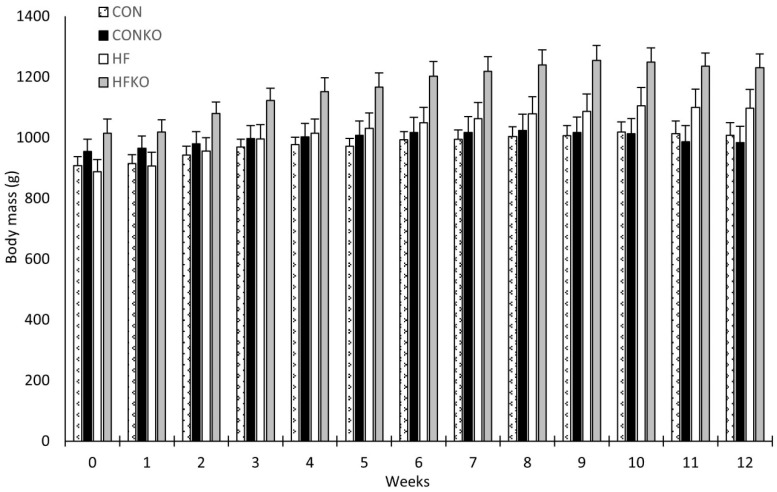
Comparison of weekly mean body mass between dietary groups. Statistically significant between-subject effects identified with greater mass noted in the HFKO group when compared to the CON group (One-way repeated measures ANOVA, *p* < 0.05). Error bars represent SEM. n = 7–8 per group. CON: control diet; CONKO: control diet with krill oil supplementation; HF: high-fat diet; HFKO: high-fat diet with krill oil supplementation.

Baseline body mass was assessed at 16 months and then measured weekly. One-way repeated measures ANOVA was conducted to identify statistically significant, between-subjects effects. Overall, body weight increased for all groups when comparing baseline to week 12: CON, CONKO, HF, and HFKO mean body weight increased by 11.0%, 3.0%, 23.6% and 21.3%, respectively. Diet did elicit statistically significant changes in body weight over time, *p* = 0.021. Tukey HSD post hoc analysis demonstrated a statistically significant overall increase in body mass in HFKO when compared to CON (*p* = 0.019). Otherwise, no differences identified between dietary groups (Figure 2).

### 2.4. Behavioral Testing

**Figure 3:**
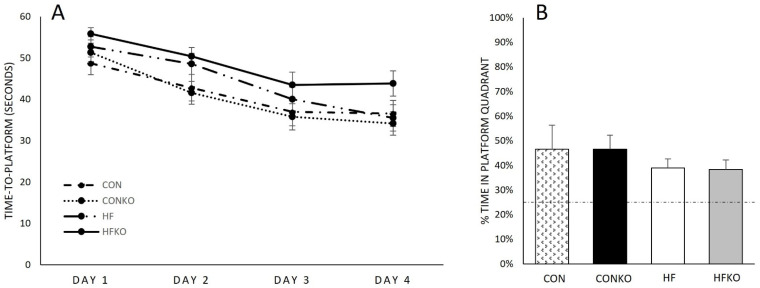
Morris water maze testing. (**A**) Time-to-platform for each dietary group on testing days 1–4. Although each group demonstrated learning with overall decreases in time-to-platform over the four testing days, diet did not induce statistically significant differences. (**B**) Morris water maze probe trial. No statistically significant differences were identified between groups when comparing the percentage of time spent in the removed platform quadrant. All groups showed a preference for the target quadrant with time spent greater than 25%. The dashed line indicates platform choice equivalent to random chance (25%). Error bars represent SEM. n = 7–8 per group. CON: control diet; CONKO: control diet with krill oil supplementation; HF: high-fat diet; HFKO: high-fat diet with krill oil supplementation.

Morris water maze time-to-platform was recorded over 4 consecutive testing days (Figure 3A). A one-way ANOVA was utilized for time-to-platform comparisons between groups. The mean time-to-platform for all four groups on day one and day four (mean seconds ± SEM) was as follows: [Day 1: CON (48.7 ± 2.8); CONKO (51.4 ± 2.3) HF (52.8 ± 2.4); HFKO (55.9 ± 9.8)]; [Day 4: CON (36.6 ± 3.2); CONKO (34.2 ± 2.8) HF (35.5 ± 3.2); HFKO (43.9 ± 3.0)]. Day 1 time-to-platform comparison was not significantly different, *p* = 0.196. Although time-to-platform improved over the duration of testing for all dietary groups, no statistically significant difference between groups was identified by day 4, *p* = 0.125. Diet did not elicit statistically significant differences in the percentage of time spent in the platform quadrant during probe trial testing, *p* = 0.642. Times from one animal were not included in data analysis due to repetitive, prolonged water submersion (Figure 3B).

### 2.5. Cytokine Concentrations

**Figure 4:**
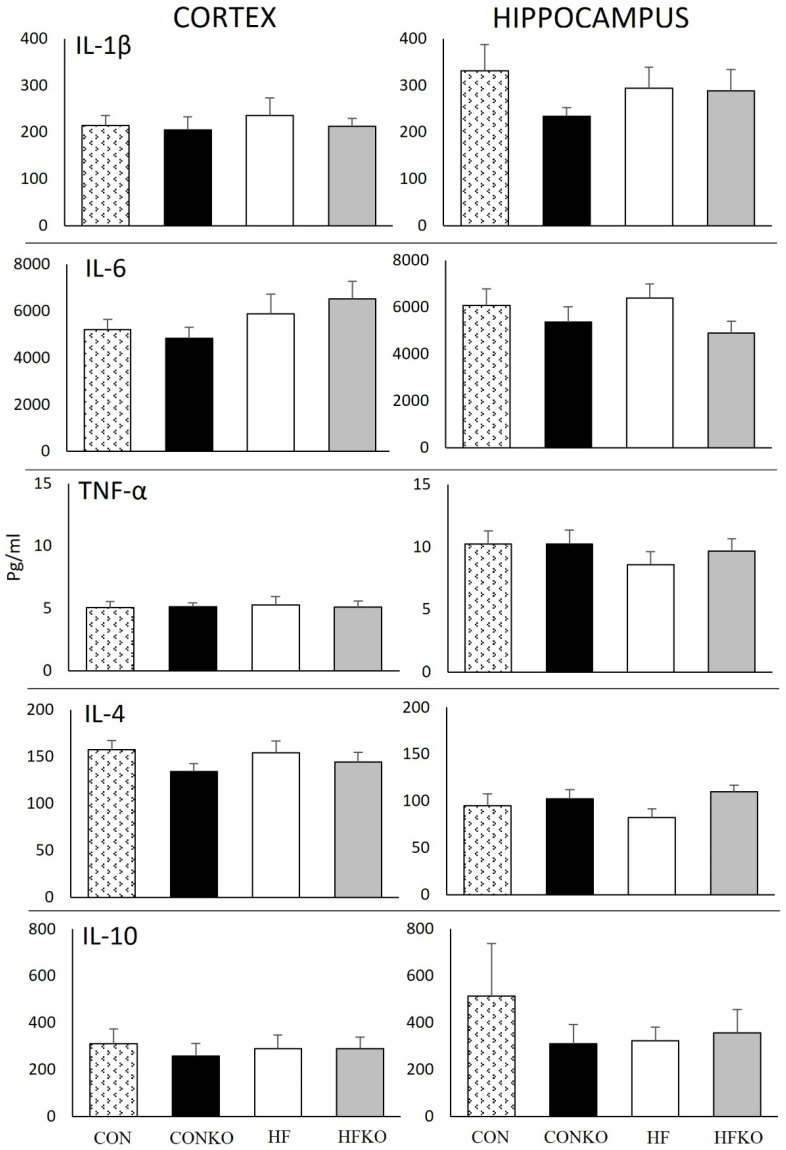
Pro-inflammatory and anti-inflammatory cytokine concentrations in the hippocampus and cortex. Error bars represent SEM. n = 7–8 per group. CON: control diet; CONKO: control diet with krill oil supplementation; HF: high-fat diet; HFKO: high-fat diet with krill oil supplementation.

Diet did not induce statistically significant differences in cortex or hippocampal cytokine concentrations (Figure 4). Cortex: [IL-1β, *p* = 0.867], [IL-6, *p* = 0.267], [TNF-α, *p* = 0.991], [IL-4, *p* = 0.400], [IL-10, *p* = 0.933]. Hippocampus: [IL-1β, *p* = 0.570], [IL-6, *p* = 0.361], [TNF-α, *p* = 0.545], [IL-4, *p* = 0.254], [IL-10, *p* = 0.703].

### 2.6. Microglia and Doublecortin Quantification

**Figure 5:**
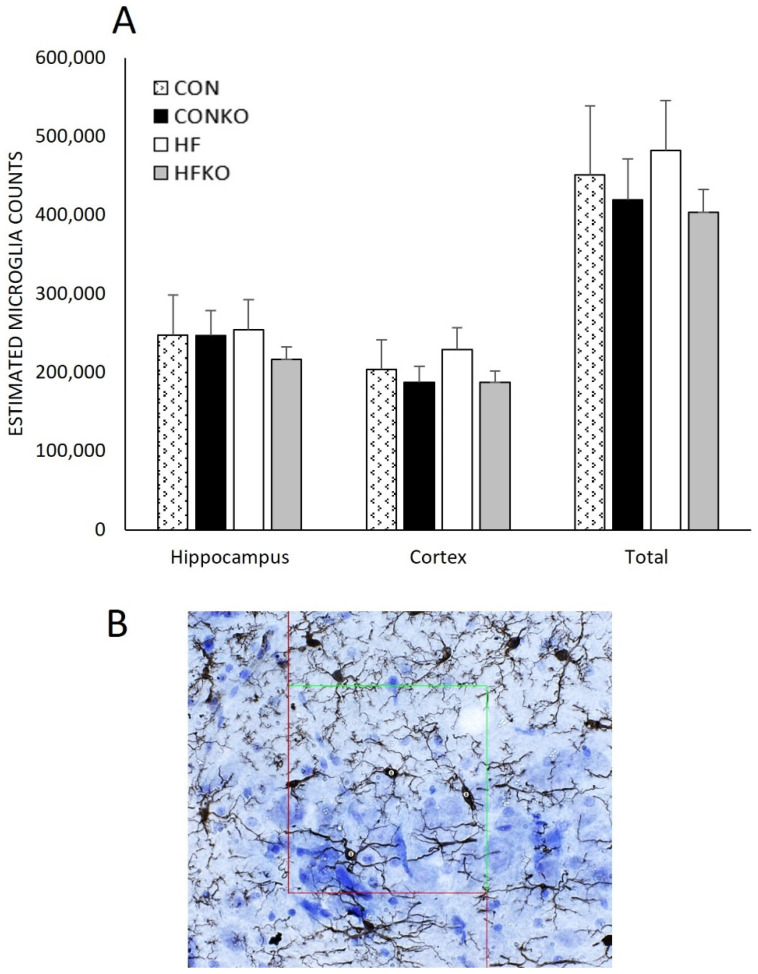
Comparison of stereological analysis of microglia between dietary groups in the hippocampus and cortex. (**A**) Quantification of microglia counts in the rat cortex, dentate gyrus and total combined. Error bars represent SEM. n = 4–6 per group. CON: control diet; CONKO: control diet with krill oil supplementation; HF: high-fat diet; HFKO: high-fat diet with krill oil supplementation. (**B**) Representative image of microglia at 6300× magnification. Ionized calcium-binding adaptor molecule 1 (Iba-1) antibody, DAB substrate, and cresyl violet staining. StereoInvestigator dissector box and markers shown.

Stereology techniques were utilized to quantify microglia in the cortex and dentate gyrus, in addition to the total, combined counts of both regions (Figure 5). Diet did not induce statistically significant differences in microglia counts: CON (n = 4), CONKO (n = 6), HF (n = 4), and HFKO (n = 5). No statistically significant differences were identified between groups: hippocampus, *p* = 0.863; cortex, *p* = 0.623; combined, *p* = 0.797.

**Figure 6:**
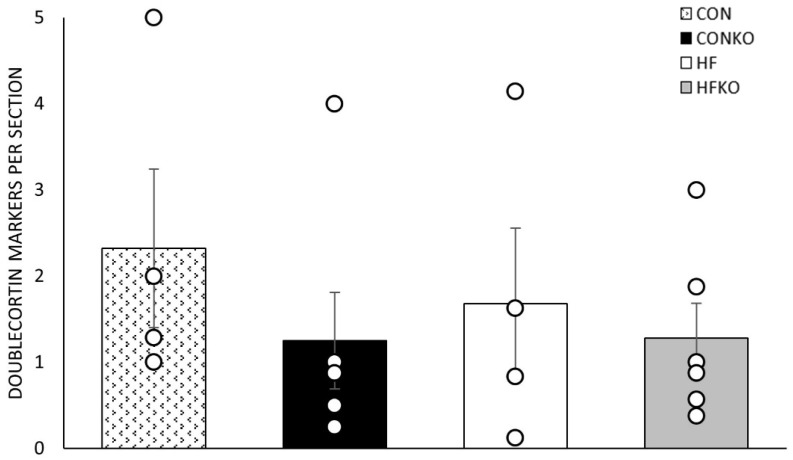
Comparison of average doublecortin markers per section in the dentate gyrus. Circle symbols are individual data points. Error bars represent SEM. n = 4–6 per group. CON: control diet; CONKO: control diet with krill oil supplementation; HF: high-fat diet; HFKO: high-fat diet with krill oil supplementation.

Doublecortin staining was used to quantify hippocampal neurogenesis in the dentate gyrus. (Figure 6). Diet did not result in statistically significant differences in neurogenesis markers between dietary groups, *p* = 0.661.

### 2.7. Synaptic Density

**Figure 7:**
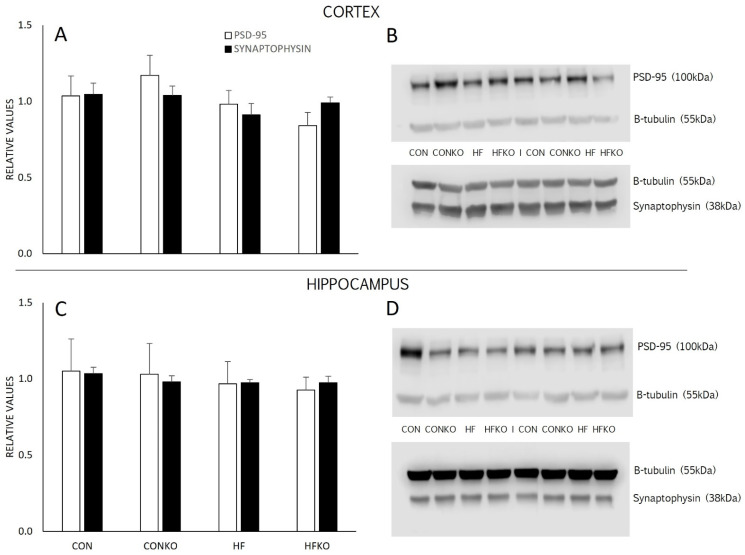
Synaptophysin (SYP) and post-synaptic density-95 (PSD-95) in the cortex and hippocampus. Western immunoblotting was utilized to measure relative concentrations of SYP (38 kDa) and PSD-95 (100 kDa) with β-tubulin loading control (55 kDa) in the cortex (**A**) and hippocampus (**C**). Errors bars represent SEM. n = 7–8 per group. Representative blots for cortex (**B**) and hippocampus (**D**). CON: control diet; CONKO: control diet with krill oil supplementation; HF: high-fat diet; HFKO: high-fat diet with krill oil supplementation.

Synaptophysin and PSD-95 relative concentrations, with β-tubulin loading protein, were assessed as markers of synaptic density (Figure 7A,C). Diet did not induce statistically significant differences in synaptic density between groups in either the cortex or hippocampus tissue samples (Cortex: PSD-95, *p* = 0.226; SYP, *p* = 0.436. Hippocampus: PSD-95, *p* = 0.948; SYP, *p* = 0.643). The lack of significant differences is evident in the representative blots (Figure 7B,D).

## 3. Discussion

This study sought to determine whether the negative consequences of aging and a HFD on learning and other brain health biomarkers could be counteracted by KO dietary supplementation. Contrary to our hypotheses, and what some studies suggest, the current dose of KO provided for this study did not enhance many of the brain parameters measured, including spatial memory, cytokine concentrations, microglia count, synaptic density, or neurogenesis. The dietary intervention did induce statistically significant differences in body weight and food consumption between select groups. The HF group consumed less food compared to the CON and HFKO groups, while body weight was significantly greater in the HFKO group when compared to CON. Moreover, HF gained the most weight while consuming the least amount of food. We observed a trend where both high-fat experimental diets (60% kcal from fats) gained dramatically more weight than the two low-fat diets (23% kcal from fats), likely due to diet composition and the large discrepancy in energy density between diets. Predictably, consumption of diets containing a greater percentage of energy-dense macronutrients contributed to substantial weight gain. Conversely, the two CON groups gained much less weight over the duration of the experiment, despite the lack of physical activity, further supporting the notion that decreasing intake of energy-dense foods plays an important role in weight management. Indeed, obesity and HFD consumption have been linked with cognitive decline. In 1990, Greenwood and Winocur [44] demonstrated that animals consuming higher amounts of saturated fats performed more poorly on learning and memory tasks. Since these early published findings, additional research has reinforced the link between obesity, cognitive impairment, and increasing risks of developing neurodegenerative disease [6,12,45,46,47,48].

Overall, in our experimental paradigm, KO did not provide benefits against aging cognitive decline with or without an HFD. These findings challenge the belief about the benefits of KO supplementation for brain health (see Andraka et al., 2020 for review [6]). Our current study also provides noteworthy information for future exploration of the efficacy of KO in combatting age and diet-induced brain changes. This study was indeed designed according to previous published research supporting the neuroprotective benefits of consuming marine-based, long-chain *n*-3 PUFAs [6]. Despite anecdotal justification, limited research demonstrates KO’s propensity to ameliorate the effects of an HFD and aging on neuroinflammation and cognition [49,50,51,52]. In fact, to the authors’ knowledge, no published studies have tested the effects of KO on adult HN or synaptic density in the hippocampus or cortex. Considering KO’s purported environmental sustainability, safety for consumption, bioavailability, and inherent neuroprotective properties, along with the research suggesting the cognitive benefits of *n*-3 PUFA supplementation, we were highly motivated to test the capacity of KO’s potential for reversing the consequences of aging and an HFD [6,53,54,55].

In our aforementioned experimental conditions, KO supplementation failed to improve the detrimental effect of an HFD and aging on cognitive and physiological brain parameters. However, methodological differences may partially explain our findings compared to previous studies that have demonstrated *n*-3 PUFAs’ benefits [32,34,52,56]. DHA, one of the primary *n*-3 PUFA components of KO and FO, and the most abundant *n*-3 PUFA in the brain, has shown some promise as a neuroprotective dietary supplement [6,57]. Debate persists surrounding optimal DHA concentrations sufficient to ameliorate the effects of aging on brain health and cognitive function. Indeed, research reporting DHA concentrations is quite varying. Heterogenous methodologies, animal breeds, pathological models, and DHA delivery modes are reported [57]. For example, two-month-old rats were tube-fed DHA at concentrations of 150, 300, or 600 mg/kg/day. Interestingly, the low-dose groups demonstrated improved spatial learning and probe trial performance, while high doses impaired performance on MWM testing, indicating that an optimally beneficial concentration of DHA exists [58]. In a pathological AD mouse model given 450 mg/kg daily via intragastric administration, no significant effects on MWM or neuroinflammatory markers was evident [59]. Further complicating analyses, DHA is often administered in a form that contains additional compounds such as *n*-6 PUFAs, short-chain *n*-3 PUFAs, EPA, choline, or ASTA, making it difficult to determine therapeutic dosages and specific benefits. One must consider that previous research exploring the benefits of *n*-3 PUFAs investigating varying animal models, transgenic species, dosages, supplementation duration, and delivery modes have produced inconsistent results. Indeed, one study found that water maze scores significantly improved over controls in 50 and 100 mg/kg/day DHA-treated aged mice, signifying DHA’s potential for improving aging related cognitive dysfunction even at lower doses [60]. Our animals were permitted food and water ad libitum; therefore, based on typical daily food intake and previous research in our lab, we estimated that milling 8% KO into experimental diets would result in DHA intake of approximately 100–150 mg/kg/day. Indeed, considering average daily food intake over the duration of the study, we administered approximately 170.3 and 137.0 mg/kg/day of DHA to the CONKO and HFKO groups, respectively, delivered in KO form. However, evidence points to DHA being less well absorbed in the blood and brain of aged rodents when compared to young ones; therefore, higher dosages may be necessary to induce therapeutic effects in aged models [61].

Moreover, we used Sprague Dawley rats as a rodent model, particularly for their potential for behavioral testing and naturally occurring neuroinflammation during aging. Several previous studies exploring the negative effects of an HFD on cognition and brain health used C57BL/6 mice, an inbred genetically uniform breed that has been suggested as more susceptible to the effects of an HFD compared to outbred rats [47,62]. In our opinion, however, using Sprague Dawley rats, which have been extensively used in various nutrition, diabetes mellitus, obesity, and aging models, leads to an improved generalizability of results, and therefore is preferable when the intent is to extrapolate results to a genetically diverse human population.

It can also be argued that increasing the duration of dietary supplementation and perhaps beginning supplementation earlier in life may have resulted in a more sizable treatment effect. The animals in this design were aged to approximately 16 months when KO was introduced. Animals were approaching “late middle-age” considering that the median life span for male Sprague Dawley rats is 25 months [63]. Since increased use of supplements and health-driven dietary modifications are more commonly implemented mid-life, the timing of experimental diet administration was intended to mimic typical human behavior. However, it is clear from our findings that our non-pharmacological intervention is not sufficient to reverse the marked effects of aging on neuroinflammation, HN, and synaptic density. Nonetheless, it is also clear that many parameters of the current design could have affected the outcome of this study, and therefore additional experimentation should be performed to more definitively understand the impact of dietary KO during aging. We chose an experimental HFD consisting of 60% kcal from fat to increase the likelihood of a dramatic diet-induced effect. This decision was based on preliminary experiments in our lab as well as previously published research from other laboratories [47,48]. A “Western diet” consisting of approximately 40% kcal did not result in marked brain inflammation or measurable deficits in hippocampal cognitive function in C57BL/6 mice and Wistar rats [46,47,48]. Conversely, the same authors reported that a diet consisting of 60% calories from fat resulted in significant increases in all target cytokines. These previous works support our hypothesis that an HFD would likely induce adverse brain changes. However, significant variability in dietary duration (12, 16, 18, 20, 21, 36 weeks), animal model (C57BL/6 mice, Long Evans rats, Sprague Dawley, Wistar rats), and age at diet initiation (1, 2, 8, 12, 21 months of age) is noted in the literature [23,33,46,47,48,64,65]. Perhaps prolonging the duration and/or initiating experimental diets earlier in life could result in more profound changes. Nonetheless, the clear lack of effects in our paradigm does not suggest that large benefits could be observed even with longer duration of supplementation.

The ratio of *n*-6 to *n*-3 in our experimental diets is also worthy of discussion. Table 1 provides complete dietary formulations as well as lipid profiles of KO, SO and lard. *n*-3 PUFA and *n*-6 [e.g., arachidonic acid (AA)] are precursors to potent lipid mediator signaling molecules, termed eicosanoids, which have important roles in regulating inflammation [66]. Eicosanoids derived from *n*-6 are generally considered pro-inflammatory. Unfortunately, *n*-6 fatty acids account for the majority of PUFA consumed in Western diets, with *n*-6/*n*-3 ratios increasing from 1:1 during early humankind to as high as 20:1 in contemporary Western diets [67]. Consequently, dietary *n*-6/*n*-3 ratios can have significant health implications. Indeed, elevated *n*-6/*n*-3 PUFA ratios are associated with increases in pro-inflammatory cytokine levels, cognitive decline, and elevated incidence of dementia [68,69]. Conversely, lower *n*-6/*n*-3 ratios suppress neuroinflammation, improve hippocampus-dependent spatial memory, enhance learning on virtual navigation tasks, and improve overall cognitive status [37,68,69,70]. Our research aimed to test the capacity of KO, with its combination of beneficial *n*-6/*n*-3 PUFA ratios and additional known anti-inflammatory and antioxidant compounds including ASTA and choline, to offset the effects of aging and an HFD on brain health. When comparing lipid profiles of KO, SO, and lard (Table 1d), our dietary composition should have been sufficient to detect the effects of these “active” components of KO, mainly DHA and EPA. Indeed, SO contains higher levels of linolenic acid, a known precursor to DHA; however, this conversion is inefficient and negligible, and thus not likely to elevate brain DHA levels in CON groups [71,72]. Our experimental diets are unquestionably nutritionally imbalanced. However, considering the societal increase in the consumption of diets with higher *n*-6/*n*-3 ratios, a secondary aim of our methodology was to identify differences in brain health biomarkers due to significant variations in *n*-6/*n*-3 ratios. For example, the two CON diets had identical nutritional formulations except for the KO and SO exchange (Table 1a–c). Indeed, both CON diets were low-fat. However, *n*-6/*n*-3 ratios were reduced in CONKO, while in CON they were dramatically elevated due to KO being replaced by SO, which is composed of over 50% *n*-6. Likewise, when comparing HF experimental diets. The primary aim of our study was to determine whether KO could counter the effects of aging and aging with a high-fat diet; however, the imbalances in *n*-6/*n*-3 ratios when comparing the CON and HF groups are noteworthy. KO supplementation and the beneficial suppression of *n*-6/*n*-3 dietary ratios was also unable to improve learning or brain health physiological markers in our model when compared to diets with unhealthy PUFA ratios.

Quantification of microglia was not significantly different between dietary groups when comparing counts in the hippocampus and cortex (Figure 5A). Although microglia are highly dynamic, age-related changes in microglia mobility and adaptability have been implicated in neurodegenerative disease processes. Indeed, microglia’s capacity for mobility and motility is diminished with age, while young microglia more vigorously respond to injury-associated signaling [17,73,74,75]. We did not measure mobility directly, but perhaps the diminished responsiveness of aged microglia contributed to the absence of significant changes due to an HFD and the apparent lack of anti-inflammatory effects of KO. Synaptic pathology and suppression of adult hippocampal neurogenesis are associated with learning and memory deficits, which are commonly identified as indicators of neurodegenerative disease processes [76,77]. Indeed, an HFD and typical aging negatively impact synaptic junction integrity and density and impair performance in various cognitive tasks such as object recognition [78,79,80]. Numerous pharmacological and non-pharmacological treatments promote beneficial increases in dendritic spine numbers and synaptic density in the hippocampus, while evidence on KO is lacking [81,82]. Moreover, mounting evidence supports the link between cognitive impairment and impaired neurogenesis, while diminished markers of neurogenesis are viewed as precursors to neurodegenerative disorders [83,84]. We hypothesized that diets enriched with KO would benefit synaptic density and neurogenesis; however, this was not evident in our findings (Figure 6 and Figure 7). Again, to the authors’ knowledge, no current research exists exploring the effects of KO on synapse-associated proteins or neurogenesis, further supporting the novel nature of this investigation and its usefulness in complementing the current body of knowledge in this field.

## 4. Materials and Methods

### 4.1. Materials

Unless otherwise noted, all chemicals were purchased from Thermo Fisher Scientific (Hanover Park, IL, USA) or Sigma-Aldrich (St. Louis, MO, USA). Rat diets were manufactured by Inotiv (Indianapolis, IN, USA) and krill oil was provided by Aker Biomarine (Lysaker, Norway). Reagents and apparatus for Western immunoblotting were obtained from Bio-Rad Laboratories (Hercules, CA, USA).

### 4.2. Animal Care and Dietary Groups

The procedures performed were approved by Central Michigan University’s Institutional Animal Care and Use Committee, Naveen Sharma P.I., protocol #19-08. Thirty-six male Sprague Dawley rats were purchased at approximately 6 months of age (Charles River, Hollister, CA, USA). The animals were housed individually and maintained in a 12:12 h light–dark cycle in a specific pathogen-free environment. Animals were fed standard chow (#2018, Inotiv Teklad Diets, Madison, WI, USA) until 16 months of age and were then randomly assigned to one of four dietary groups: control (CON); control with KO supplementation (CONKO); high-fat diet (HF); and high-fat diet with KO supplementation (HFKO) (Table 1a–d). HF diets contained 5.1 kcal/g compared to 3.9 kcal/g for the two low-fat groups, while 8 g/100 g of KO or soybean oil was administered to the KO and non-KO groups, respectively. Animals were exclusively fed assigned diets for twelve weeks and were allowed access to food and water ad libitum. Food consumption and body mass were recorded weekly.

### 4.3. Behavioral Testing

Water pool testing was performed similarly to previous work carried out by our group [85]. Briefly, 19.0-month-old rats were tested utilizing a 170 cm diameter, 20 °C water maze with white walls. The pool was surrounded by white curtains with multiple visual cues. The circular escape platform was 10 cm in diameter, present in a constant location and submerged about 2.5 cm below the surface of the water. Rat movement was monitored using Ethovision XT14 (Noldus, Leesburg, VA, USA). Each rat performed three training blocks per day, two trials per block, for 4 days (24 trials total) with a 60 m inter-block interval. On day 4 of testing, an additional probe trial was added, whereas the submerged platform was removed, and time spent in the quadrant where the platform had been located was documented. On each trial, the rat was randomly released into the water from one of six locations spaced evenly at the side of the pool. After finding the submersed platform or swimming for 60 s, the animal remained on the platform for 30 s.

### 4.4. Tissue Collection

Following behavioral testing, rats were anesthetized under isoflurane at a rate maintained between 1% and 3% and oxygen at 1 L/min, then quickly sacrificed by decapitation at a mean age of 19.0 ± 1.6 months. Brain samples were quickly removed and divided into hemispheres. The cortex and hippocampus were isolated from one hemisphere, rapidly frozen in liquid CO_2_, and stored at −80 °C for later use in cytokine concentrations and synaptic density analysis. The remaining brain hemisphere was immediately submersed in 4% paraformaldehyde (PFA) for 7 days, and then stored in 1x phosphate-buffered saline (PBS), pH 7.4, for approximately 1–2 weeks prior to sectioning for stereological analysis.

### 4.5. Protein Analysis

Approximately 50 mg each of hippocampus and cortex tissue was transferred to respective microfuge tubes and homogenized in ice-cold Tissue Protein Extraction Reagent buffer (#78510; Thermo Fisher Scientific) using a TissueLyser LT (#85600; Qiagen, Germantown, MD, USA) at 30 cycles for 6 min. Homogenates were rotated for 1 h at 4 °C to break up the lipid bilayer and intracellular vesicles, and then centrifuged (15,000 rpm) for 15 min at 4 °C to remove insoluble material. The protein concentration of homogenates was determined by means of a BCA protein assay kit (#23227; Thermo Fisher Scientific). Cytokines including IL-1β, IL-6, TNF-α, IL-4 and IL-10 were detected using a commercially available multiplex kit (#RECYTMAG-65PMX27BK, EMD Millipore, Billerica, MA, USA) utilizing magnetic bead-based multiplex technology (Luminex, Austin, TX, USA). Duplicate sample analysis was completed to assure consistent and reliable protein concentration measurements. Protein concentrations were reported in picograms per milliliter (pg/mL).

Tissue homogenates were mixed with 2x Laemmli sample buffer (LSB) volumes based on 40 μg protein concentrations, boiled for 5 min at 100 °C, and then flash spun. The samples were separated using the SDS-PAGE (Sodium dodecyl sulfate-polyacrylamide gel electrophoresis) process with 10% prefabricated polyacrylamide gels (BioRad, Hercules, CA, USA) before being transferred to nitrocellulose membranes. Membranes were rinsed in TBS (Tris-buffered saline) for 5 min at room temperature (RT) and then blocked in 5% BSA (Bovine serum albumin)/TBST (Tris-buffered saline with Tween 20) for 1 h at RT. Membranes were rinsed in TBST for 5 min at RT and then transferred to the appropriate primary antibody and loading protein mixed with 5% BSA/TBST (1:1000) overnight at 4 °C. The primary antibodies utilized to assess synaptic density in cortex and hippocampus samples were synaptophysin (#ab8049, Abcam, Boston, MA, USA), post-synaptic density-95 (#MAB1596, Millipore Sigma, Burlington, MA, USA) and beta-tubulin (#T5201, Millipore Sigma, Burlington, MA, USA) loading protein. Membranes were then washed 3 times for 5 min in TBST at RT and incubated with anti-mouse, HRP-linked secondary antibody (1:10,000; #7076, Cell Signaling, Danvers, MA, USA) for 1 h at RT. Blots were then rinsed 3 times for 5 min in TBST then 2 times for 5 min in TBS at RT. The blots were incubated for 5 min at RT with an enhanced-chemiluminescence (ECL) Western blotting substrate (cat#34075, Thermo Fisher Scientific) to visualize protein bands. Total detected PSD-95, synaptophysin and loading proteins bands were quantified by means of densitometry and normalized using the average density of all bands on individual membranes (ProteinSimple, Santa Clara, CA, USA).

### 4.6. Section Preparation and Stereology

After immersion in PFA, free-floating sagittal sections (30 µm) were obtained using a Vibratome VT1000S (Leica Biosystems, Deerpark, IL, USA). Sections were prepared using the following primary antibodies for microglia and doublecortin: ionized calcium-binding adaptor protein-1 (Iba-1; 1:1000; #ab178846, Abcam, Boston, MA, USA) and doublecortin (DCX; 1/1,000; #ab18723, Abcam, Boston, MA, USA). After quenching endogenous peroxidase activity and blocking nonspecific binding, the sections were incubated (4 °C) overnight with primary antibodies. Thereafter, the sections were incubated for 2 h (22 °C) with the goat anti-rabbit secondary antibody IgG-Biotin (1/5000; #4030-08, Southern Biotech, Birmingham, AL, USA). Sections were then incubated for 1 h (22 °C) with Vectastain Elite ABC avidin-biotinylated horseradish peroxidase complex (Vector, Burlingame, CA, USA). After washing again in TBS, the sections were incubated with 10X Ni-DAB reaction mix (#34065, Thermo Fisher Scientific) serving as a chromogen. The reaction was stopped by washing the sections with TBS. Sections were mounted on slides, air-dried, and cover slipped with Permount (Thermo Fischer Scientific). The location of immunohistochemically defined cells was examined by means of light microscopy. Quantification of microglia in the hippocampus and cortex was assessed with StereoInvestigator imaging software (Version 2022.2.1, MBF Bioscience, Williston, VT, USA), an optical fractionator probe, and systematic random sampling (SRS) and reported as the estimated population mean using section thickness. Live microglia counting parameters were 630× magnification, a counting size frame of 65 μm × 65 μm, and an SRS layout grid size of 550 × 550. Conversely, DCX was quantified by live counting in the dentate gyrus at 20× magnification.

### 4.7. Statistical Analysis

Statistical software (IBM SPSS Statistics, Version 28.0, IBM Corp., Armonk, NY, USA) was utilized for descriptive statistics, analysis of variance (ANOVA), and post hoc analyses. All error bars in figures represent the SEM. One-way repeated measures ANOVA was utilized for comparisons of weekly food consumption and body mass. One-way ANOVA was utilized for the comparison of cytokine concentrations, microglia, and DCX quantification and Morris water maze testing times. Tukey HSD post hoc analysis was completed when indicated. *p* < 0.05 was deemed statistically significant for all analyses. Central Michigan University Statistical Consulting Center provided guidance on statistical analysis.

## 5. Conclusions 

In the current study design, KO supplementation did not counteract the consequences of aging and a high-fat diet on spatial learning, neuroinflammation, microglia quantification, synaptic density, or neurogenesis in an aged rat model. Considering the many variables in aging studies that can influence the outcome of our conclusions (modification of diet parameters, length of diet administration, age of the animals when the diet is initiated, age of the animals at the end of the protocol, housing conditions, etc.) we can surmise that in our experimental paradigm, the benefit of KO supplementation is minimal. Based on these findings, we aim to design future studies that continue to modify these variables, allowing us to make more definitive suggestions about KO supplementation for the aging brain. Forthcoming research will indeed continue to emphasize aging models; however, experimental diets will be initiated earlier in the lifespan, such as 8 or 12 months of age, and the duration will be prolonged. Additionally, experimenting with varying amounts of dietary KO will aid in our attempts to identify an optimal therapeutic KO dose.

## Figures and Tables

**Table 1:** (**a**) Dietary groups: Percentage by weight of varying fats, carbohydrates, and protein. CHO: carbohydrates; PRO: protein; KO: krill oil; SO: soybean oil. The CON, CONKO, HF, and HFKO groups were fed experimental diets for 12 weeks. (**b**) Dietary groups: Percentage kcal from fats, carbohydrates, and protein and energy density in kcal per gram of food. CHO: carbohydrates; PRO: protein; FAT: all fats including krill oil, soybean oil, and lard. (**c**) Experimental diet formulations (g/100 g). (**d**) Lipid profiles of krill oil, soybean oil, and lard (g/100 g).

(**a**)					
**Dietary Groups**	**CHO**	**PRO**	**LARD**	**KO**	**SO**
Control (CON)	57.2	18.6	2.0	0	8.0
Control with Krill oil (CONKO)	57.2	18.6	2.0	8.0	0
High fat (HF)	27.6	23.5	26.0	0	8.0
High fat with Krill oil (HFKO)	27.6	23.5	26.0	8.0	0
(**b**)					
**Dietary Groups**	**CHO**	**PRO**		**FAT**	**kcal/g**
Control (CON)/ Control with krill oil (CONKO)	57.9	18.8		23.3	3.9
High fat (HF)/ High fat with krill oil (HFKO)	21.5	18.3		60.2	5.1
(**c**)					
	**CON**	**CONKO**		**HF**	**HFKO**
Krill oil	0	8		0	8
Soybean oil	8	0		8	0
Lard	2	2		26	26
Casein	21	21		27	27
L-cystine	0.3	0.3		0.4	0.4
Corn starch	40.5	40.5		0	0
Malodextrin	10	10		16.5	16.5
Sucrose	9	9		9	9
Cellulose	3.7	3.7		6.6	6.6
Mineral mix, AIN-93G-MX	3.5	3.5		4.5	4.5
Calcium phosphate, dibasic	0.2	0.2		0.3	0.3
Vitamin mix, AIN-93-VX	1.5	1.5		1.9	1.9
Choline bitrartrae	0.3	0.3		0.3	0.3
(**d**)					
	**Krill Oil**	**Soybean Oil**		**Lard**	
Eicosapentanoic acid (20:5)	15	─		─	
Docosahexaenoic acid (22:6)	7	─		─	
Palmitic acid (16:0)	23	10		23	
Stearic acid (18:0)	1	4		13	
Oleic acid (18:1)	18	18		39	
Linoleic acid (18:2)	3	55		18	
Linolenic acid (18:3)	1	13		1	

## Data Availability

The raw data supporting the conclusions of this article will be made available by the authors upon request.
